# Ferroptosis, pathogenesis and therapy in AS co-depression disease

**DOI:** 10.3389/fphar.2025.1516601

**Published:** 2025-02-24

**Authors:** Yulong Zhao, Peng Ren, Qiang Luo, Xue Li, Xinyi Cheng, Youliang Wen, Xiaoyun Wu, Junjie Zhou

**Affiliations:** ^1^ School of Rehabilitation Medicine, Gannan Medical University, Ganzhou, Jiangxi, China; ^2^ School of Basic Medicine, Gannan Medical University, Ganzhou, Jiangxi, China; ^3^ Key Laboratory of Prevention and Treatment of Cardiovascular and Cerebrovascular Diseases of Ministry of Education, Gannan Medical University, Ganzhou, China; ^4^ Ganzhou Key Laboratory of Rehabilitation Medicine, Ganzhou, Jiangxi, China

**Keywords:** ferroptosis, atherosclerosis, depression, comorbid, pathogenesis, therapy

## Abstract

Atherosclerosis (AS)-related cardiovascular disease and depression are often comorbid, with patients with cardiovascular disease facing an increased risk of depression, which worsens AS. Both diseases are characterized by oxidative stress and lipid metabolism disorders. Ferroptosis, a form of cell death characterized by iron overload and harmful lipid peroxide accumulation, is found in various diseases, including AS and depression. Consistent with the iron deposition and lipid peroxidation (LPO) that characterize the ferroptosis mechanism, disturbances in iron and lipid metabolism are also crucial pathogenic mechanisms in AS and depression. The comorbid mechanisms are complex, posing challenges for clinical treatment. Chinese herbs hold significant potential owing to their multi-target pharmacological effects. Therefore, this review aims to investigate iron overload, LPO, and ferroptosis across various cell types, the shared pathogenesis of AS and depression with ferroptosis, and research on Chinese herbal medicine targeting ferroptosis in the treatment of anti-AS co-depression. This provides a comprehensive understanding of AS co-depression disease from the perspective of ferroptosis.

## Graphical Abstract

**Figure:**
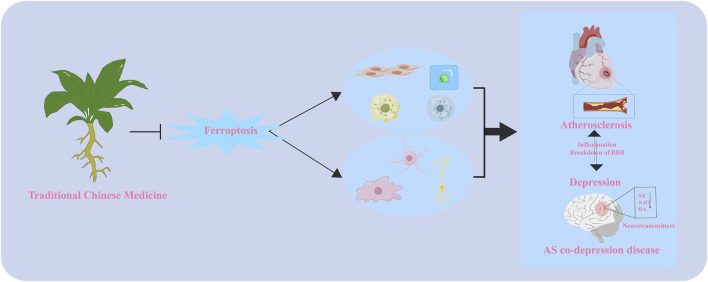

## 1 Introduction

Atherosclerosis (AS), the pathological basis of cardiovascular disease, is a threat to human life and health. According to the World Health Organization, cardiovascular disease accounts for up to 17.9 million deaths annually, representing approximately 30% of global mortality (Herrington et al., 2016; Tsao et al., 2023). AS is a chronic inflammatory condition characterized by endothelial cell damage, lipid accumulation within arterial walls, smooth muscle cell growth, and foam cell formation. Depression, a mood disorder characterized primarily by persistent feelings of sadness and anhedonia, arises from diverse causes. It has become a significant public health concern, with a global prevalence rate of 4.4% (Monroe and Harkness, 2022; WHO, 2017). Extensive research has established a strong relationship between depression and cardiovascular disease (Hare et al., 2014; Kawada, 2017; Li C. et al., 2020). Meta-analyses highlight the profound detrimental effect of depression on the development and outcomes of cardiovascular disease (Krittanawong et al., 2023). Similarly, with a co-prevalence of 17%–47%, depression is highly prevalent among individuals with cardiovascular disease (Mavrides and Nemeroff, 2013). Data from a recent U.S. health survey reveals a significant positive connection between adult depression and plasma AS index (Ye et al., 2024). Furthermore, evidence from multi-omics studies (Huang et al., 2023), Mendelian randomization analyses (Lei et al., 2023; Shakt et al., 2024), and the vascular depression hypothesis (Alexopoulos et al., 1997) underscores the close relationship between AS and depression. Despite growing evidence that they occur simultaneously and affect each other, the mechanisms linking AS and depression are still poorly understood, which complicates effective treatment strategies.

The interaction mechanism between AS and depression is highly complex, involving multiple potential mechanisms, including inflammation (Chrysohoou et al., 2018; Halaris, 2017), hypothalamic-pituitary-adrenal axis dysfunction (Gu et al., 2012), endothelial dysfunction (Chrysohoou et al., 2018; Paranthaman et al., 2012), immune responses (Mousa et al., 2022), autonomic nervous system dysfunction (Pizzi et al., 2010), and intestinal flora and metabolite dysfunction (Liao et al., 2023). Lipid metabolism disorders play a crucial role, with numerous studies confirming alterations in lipid composition in the plasma and brain of patients with AS and depression. High-fat diets disrupt brain lipid metabolism and promote neuroinflammation, increasing depression risk (Wang A. et al., 2023). Mendelian randomization analysis reveals a causal connection between altered triglyceride, cholesterol levels, and depressive phenotypes (So et al., 2021). Elevated oxidized low-density lipoprotein (Ox-LDL) levels contribute to the development of major depression by inducing neuroinflammation (Almulla et al., 2023).

Additionally, a mouse model of AS-related depression was developed using APOE^−/−^ mice to investigate the underlying pathogenesis. Differential phosphatidylethanolamine (PE) levels in the hippocampus and prefrontal cortex of the model mice are closely linked to ferroptosis (Hu et al., 2022). PE, the second most abundant phospholipid in living organisms, plays a crucial role in depression. Disruptions in PE metabolism can impair mitochondrial activity in the brain, contributing to depressive symptoms (Modica-Napolitano and Renshaw, 2004). Furthermore, individuals with seasonal depression exhibit altered plasma PE levels (Otoki et al., 2017). The role of ferroptosis in AS has also been extensively demonstrated (Fang et al., 2023). Stockwell further highlights its significance in cardiovascular and neurodegenerative diseases (Stockwell, 2022). Therefore, the study of AS co-depression diseases from the perspective of ferroptosis has a sufficient theoretical and experimental basis.

## 2 Method

This review aims to summarize the mechanism of ferroptosis in the pathogenesis of atherosclerotic comorbid depression and to highlight the research progress on the use of Chinese medicine in treating this condition by targeting ferroptosis. Keywords included the following: “ferroptosis,” “iron death,” “atherosclerosis,” “depression,” “cardiovascular and cerebrovascular disease,” “major depressive disorder,” and “Traditional Chinese medicine.” Searches were conducted using multiple online databases, including Google Scholar, PubMed, Scopus, Embase, Web of Science, and ScienceDirect, for English-language journal reports and articles published up to October 2024. References were manually screened from the extracted articles.

## 3 Overview of ferroptosis

Cell death is fundamental to organismal growth, development, senescence, and death, involving mechanisms such as apoptosis, necrotic apoptosis, autophagy, cellular pyroptosis, and necrosis, each with distinct characteristics (Moujalled et al., 2021). Ferroptosis, a regulated form of cell death distinct from apoptosis, was introduced by Dixon in 2012 (Dixon et al., 2012) and defined by the Cell Death Commission in 2018 (Galluzzi et al., 2018). The condition is characterized by redox imbalance and iron overload, with affected cells exhibiting smaller mitochondria, increased membrane density, and reduced cristae, while the nucleus shows minimal changes (Li J. et al., 2020). Cellular alterations include increased lipid peroxides, elevated reactive oxygen species (ROS), and reduced Glutathione Peroxidase 4 (GPX4) levels (Chen et al., 2021b). Ferroptosis affects tumor development and various disorders, including neurological, ischemic, and cardiovascular conditions (Fang et al., 2023) and depression (Chen et al., 2023). Furthermore, ferroptosis is regulated by various processes and small molecules, including iron metabolism, lipid peroxidation (LPO), the XC-GSH-GPX4 axis, ferroptosis suppressor protein 1 (FSP1), coenzyme Q10 (CoQ10)-NAD(P)H pathway, guanosine triphosphate cycloheximide hydrolase 1, tetrahydrobiopterin (BH4)-dihydrofolate reductase pathway, autophagy, mitochondrial function, among others (Chen et al., 2021a; Song Y. H. et al., 2023).

## 4 Main mechanisms of ferroptosis

Ferroptosis primarily arises from the excessive accumulation of ROS on the cell membrane owing to metabolic dysfunction. Lipid peroxides and their metabolites, such as malondialdehyde (MDA) and 4-hydroxynonenal, damage proteins, membrane integrity, and DNA structure, leading to cell membrane hyperpermeability and ferroptosis (Lei et al., 2022). Lipid peroxide accumulation results from enzymatic and non-enzymatic processes (Rochette et al., 2022). Polyunsaturated fatty acids (PUFA) are converted into reactive lipid peroxides by fatty acid enzymes and through the Fenton reaction, which is driven by iron accumulation from disrupted iron metabolism.

### 4.1 Enzymatic pathway of ferroptosis

#### 4.1.1 ACSL4/LPCAT3/LOX lipid metabolism pathway

Among the three families of lipid oxidases, lipoxygenase is crucial to the enzymatic processes driving ferroptosis. The other two families are cyclooxygenase and cytochrome P450. Lipoxygenase converts free PUFA into lipid oxides, which significantly affects iron redox reactions (Yang et al., 2016). PUFA oxidation-specific nonheme iron-containing enzymes are known as LOXs (Maheshwari, 2023). PE, derived from adrenoic acid (AdA) and arachidonic acid (AA), are key substrates for LPO. Metabolic abnormalities in PE directly influence lipid metabolic pathways involved in ferroptosis (Kagan et al., 2017).

The principal cause of ferroptosis is linked to the peroxidation of PUFAs (Gan, 2022) and PE (Amoscato et al., 2023), while monounsaturated fatty acids inhibit ferroptosis. Ferroptosis is caused by PE-AA/AdA-OOH, not by other phospholipid (PL)-OOH forms (Lei et al., 2019). The oxidation process of PE requires the involvement of ACSL4 (He et al., 2022) and LPCAT3 (Miura et al., 2021). PUFAs, particularly AA and adrenergic acid, are susceptible to oxidation owing to the weak C-H bonds in their diallyl group (Kagan et al., 2017). The LPO process proceeds as follows: Free AA/ADA is catalyzed by ACSL4 to form AA/AdA-CoA derivatives (Zhang H. L. et al., 2022). These AA/AdA-CoA derivatives are then synthesized with PE in the cell membrane by LPCAT3, producing AA/AdA-PE (Doll and Conrad, 2017). Finally, AA/AdA-PE undergoes oxidation through enzymatic and non-enzymatic pathways. The enzymatic pathway involves LOXs, while the non-enzymatic pathway is driven by hydroxyl radicals generated through the Fenton reaction. This ultimately leads to harmful lipid peroxide formation (Wang et al., 2021c).

The LPO process highlights ACSL4, LPCAT3, and LOXs as important targets for regulating ferroptosis. For instance, berberine inhibits endothelial ferroptosis and AS by suppressing ACSL4 expression (Hong et al., 2024). Xiaoyao San mitigates lipid metabolism disorder and depression in mice by inhibiting ACSL4 (Jiao et al., 2021). Myeloid LPCAT3 deficiency disrupts AA homeostasis in the liver, leading to metabolic imbalances and triglyceride accumulation (Bourgeois et al., 2021). Additionally, LOX inhibition reduces neuroinflammation and LPO by inhibiting inflammasome activation (Cakir-Aktas et al., 2023).

#### 4.1.2 System Xc -/GSH/GPX4 pathway

Ferroptosis is mainly induced by inhibiting glutathione production, with the System Xc -/GSH/GPX4 pathway playing a key role in preventing LPO. The Xc-system facilitates intracellular cysteine (Cys) absorption, which is converted into glutathione. While the Xc-system is considered the primary source of Cys, its inhibition can also lead to Cys production through the sulfur transfer pathway. Both pathways help maintain intracellular Cys levels. However, Xc-system inhibition results in glutathione and Cys deficiencies, triggering ferroptosis (Li F. J. et al., 2022). This underscores the pivotal role of the Xc-system in preserving intracellular redox balance.

GPX4, also known as phospholipid hydroperoxide glutathione peroxidase, consists of approximately 170 amino acids and has a molecular weight of about 19 kDa. Numerous GPX family members, including GPX1–GPX8, are found in mammals (Nishida Xavier da Silva et al., 2022). However, only GPX4 scavenges membrane lipid hydroperoxides. This feature is attributed to its distinct amino acid composition and spatial arrangement. GPX4 utilizes reduced GSH as a critical substrate and oxidizes it to GSSH while converting PLOOH to fatty alcohol to suppress the LPO process (Liu Y. et al., 2023). Targeted metabolomics reveals that GPX4 overexpression and knockdown influenced the lethality of 12 ferroptosis inducers, highlighting the role of Gpx4 in ferroptosis regulation (Lou et al., 2024). RAS-selective lethal 3 (RSL3) inhibits GPX4 by covalently binding to it, leading to lipid peroxide accumulation and ferroptosis induction. Glutathione deprivation converts GPX4-catalyzed peroxides to alcohols, leading to Cys deficiency, further inactivating GPX4 and promoting ferroptosis (Ursini and Maiorino, 2020). Targeting GPX4 can improve AS (Yang et al., 2024b) and depression (Qian et al., 2023).

#### 4.1.3 Ferroptosis suppressor protein 1(FSP1) -coenzyme Q10 (CoQ10)-NADPH pathway

The FSP1-CoQ10-NAD(P)H pathway is crucial in ferroptosis prevention, alongside the GPX4 pathway, the main defense mechanism. Apoptosis-inducing factor mitochondria-associated 2 (AIFM2), formerly FSP1, mitigates ferroptosis owing to GPX4 deletion. Ubiquinone (CoQ10) mediates this inhibition. FSP1 uses NAD(P)H, while the reduced form of ubiquinone captures lipid peroxyl radicals that cause LPO. Pharmacologically targeting FSP1 induces ferroptosis in various cancers, synergizing effectively with GPX4 inhibitors (Koppula et al., 2022). The FSP1-CoQ10-NAD(P)H pathway, alongside glutathione and GPX4, functions as an independent parallel mechanism to mitigate ferroptosis and PL peroxidation. In the absence of GPX4, FSP1 effectively inhibits PL peroxidation and ferroptosis by using NAD(P)H to convert oxidized ubiquinone into ubiquinol (Doll et al., 2019).

In this pathway, CoQ10 is crucial, and the inhibition of its synthesis leads to increased LPO (Arslanbaeva et al., 2022). CoQ10 is primarily synthesized in the mitochondria, though its exact origin remains unclear. In the absence of GPX4 activity, dihydroacetate dehydrogenase inhibits ferroptosis in mitochondria by enhancing CoQH2 production (Mao et al., 2021). Moreover, BH4 prevents ferroptosis by producing CoQH2, inhibiting LPO, and scavenging free radicals (Zhang G. et al., 2023). Targeting FSP1 can improve AS (Xie et al., 2022) and depression (Wu X. et al., 2024).

#### 4.1.4 Mevalonate (MVA) pathway

The MVA pathway is a key mechanism for inhibiting ferroptosis through interactions with the GSH-GPX4 and FSP1-CoQ10-NADPH pathways. It generates isoprenoid chemicals from acetyl coenzyme A. Acetyl coenzyme A condenses to form 3-hydroxy-3-methylglutaryl coenzyme A (HMG-CoA), which is then converted to MVA by HMG-CoA reductase. MVA is further metabolized into isoprenoid compounds such as gibberellin A, cholesterol, and isopentenyl pyrophosphate (IPP) (Lasunción et al., 2022). IPP is an intermediary molecule involved in ferroptosis regulation. Farnesyl phosphate synthetase converts IPP to farnesyl pyrophosphate (FPP), which is then converted into squalene by squalene synthase (SQS) and cyclized by squalene cyclase to produce cholesterol (Liang et al., 2023). IPP is crucial for synthesizing various biomolecules and, along with FPP, produces non-cholesterol compounds such as CoQ10 through the GGPS enzyme, which SQS inhibits (Chen et al., 2020). FIN56 induces ferroptosis by stimulating SQS and decreasing COQ10 production (Shimada et al., 2016).

Additionally, the MVA pathway is vital for GPX4 synthesis. Selenocysteine (Sec) must be incorporated into its catalytic center to enable its antioxidant function. This process requires IPP to activate isoprenyltransferase, which catalyzes the mutation of the Sec transporter RNA, facilitating GPX4 activity and maintenance (Jia et al., 2024). Consequently, FIN56 inhibits Sec integration into the catalytic subunit of GPX4, reducing its antioxidant capacity (Costa et al., 2023).

### 4.2 Non-enzymatic pathway of ferroptosis

#### 4.2.1 Iron homeostasis

Iron is crucial for living organisms, and its deficiency can cause health issues. Intracellular iron is stored in two major forms—an unstable intracellular free ferrous iron pool and an inert form bound to ferritin (Fuhrmann and Brüne, 2022). Ferrous and ferric iron are forms of free iron, and excess Fe^2^⁺ can trigger the Fenton reaction, producing ROS (Bogdan et al., 2016) that induce ferroptosis and LPO. The importance of iron homeostasis is evident as the iron chelator deferoxamine effectively suppresses ferroptosis induced by intracellular iron overload (Costa et al., 2023). Additionally, iron is a cofactor for enzymes such as cytochrome P450 oxidoreductase and lipoxygenase, which synthesize lipid peroxides. Iron absorption, efflux, and cellular availability are crucial for ferroptosis.

#### 4.2.2 Iron metabolism

Transferrin-bound trivalent iron binds to the transferrin receptor (TFR1), allowing direct cellular entry through endocytosis and converts it to ferrous iron through STEAP3. Ferrous iron is released by divalent metal transporter 1 (DMT1) and recycled into the cell membrane through TFR1 within the cellular labile iron pool. TFR1 expression is regulated by five iron-regulatory elements (IREs) in its 3′untranslated region. Under iron deficiency, iron-regulatory proteins (IRPs) bind to IREs to enhance TFR1 expression (Wang S. et al., 2024). Redox-active iron complexes within the unstable iron pool are stored in cells bound to ferritin, composed of light and heavy chain components. Ferritin stores free unstable iron. Nuclear receptor coactivator 4 (NCOA4) enhances free iron levels by binding to ferritin-heavy chains and aiding its transport through the lysosome. Moreover, iron is exported through PROM2-mediated exocytosis or the FPN1/SLC40A1 efflux pump, forming multivesicular aggregates.

#### 4.2.3 Regulation of ferroptosis by iron metabolism

**FIGURE 1:**
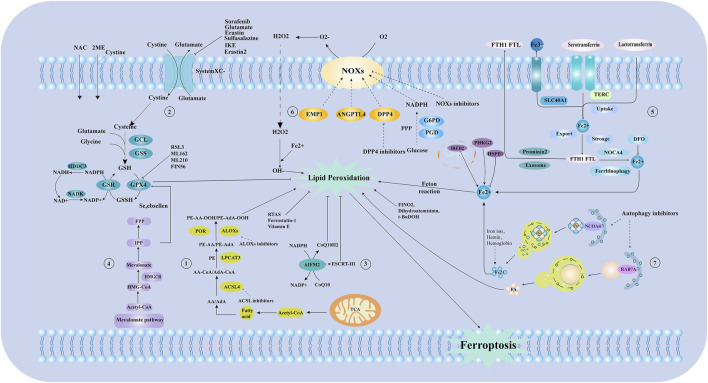
The mechanism of ferroptosis and iron metabolism. ① Lipid metabolic pathways. ② Xc-glutathione/glutathione peroxidase pathway. ③ Ferroptosis suppressor protein 1(FSP1) -Coenzyme Q10 (CoQ10)-NADPH pathway. ④ Mevalonate (MVA) pathway. ⑤ Process of iron metabolism. ⑥ Reactive oxygen species, free radical generation process. ⑦ The process of ferritin autophagy. NAC:N-Acetyl-L-cysteine; GCL: Glutamate-Cysteine Ligase; GSS: Glutathione Synthetase; GSH: Glutathione; GPX4: Glutathione Peroxidase 4; NADP: nicotinamide adenine dinucleotide phosphate; NAD: nicotinamide adenine dinucleotide; IPP: Isopentenyl pyrophosphate; FPP: Farnesyl pyrophosphate; HMG-COA:3-hydroxy-3-methyl glutaryl coenzyme A reductase; Acetl-CoA: Acetoacetyl-CoA; AA/AdA: Arachidonic acid/Adrenic acid; ACSL4:long-chain acyl-CoA synthetase 4; LPCAT3: Lysophosphatidylcholine acyltransferase 3; PE: Phosphatidyl ethanolamine; ALOXS: Arachidonate lipoxygenase; TCA: Tricarboxylic Acid Cycle; AIFM2/FSP1:Ferroptosis suppressor protein 1; EMP1: EPO mimetic peptide-1; ANGPTL4: Angiopoietin-Like Protein 4; DPP4: Dipeptidyl peptidase-4; G6PD: Glucose-6-phosphate dehydrogenase; PGD: Phosphogluconate Dehydrogenase; FTH1 FTL: Ferritin Heavy Chain 1, Ferritin Light Chain; TERC: Telomerase RNA Component; NOCA4: Nuclear Receptor Coactivator 4; DFO: Deferoxamine; RSL3: GSH peroxidase 4 inhibitors; HDDC3: HD Domain Containing 3; NADK: Nicotinamide adenine dinucleotide (NAD+) kinase; HMGCR: 3-hydroxy-3-methylglutaryl coA reductase; GSR: glutathione-disulfide reductase; 2 ME: 2-Methoxyestradiol; T-BuDOH: t-Butyl hydroperoxide; RAB7A:ras-related protein Rab-7a; ESCRT-III: Endosomal sorting complex required for transport III; CoQ10H2: Reduced coenzyme Q10; NOXs: NADPH Oxidases; SLC40A1: Solute carrier family 40 member 1; IREB2: Iron-Responsive Element Binding Protein 2; PHKG2: Phosphorylase Kinase Catalytic Subunit Gamma 2; HSPB1:Heat shock protein beta-1; PPP: pentose phosphate pathway; POR: cytochrome p450 oxidoreductase; CoQ10: Coenzyme Q10; System Xc -: Cystine/glutamate antiporter system; NCOA4:Nuclear receptor coactivator 4.

Ferroptosis can be inhibited by reducing iron uptake and using iron chelators. Conversely, transferrin removal inhibits ferroptosis, TFR1 upregulation increases susceptibility, and IRP2 inhibition confers cellular resistance (Jiang et al., 2021). Hepcidin, a liver-derived antimicrobial peptide, negatively regulates FPN1, leading to tissue iron overload and promoting ferroptosis, underscoring its crucial role in iron metabolism regulation. Typically, transferrin-bound iron predominates, but non-transferrin-bound iron (NTBI) can emerge in plasma during iron overload (Galy et al., 2024). NTBI comprises low molecular weight iron forms that can be absorbed and damage organs, unlike transferrin-bound iron. In summary, LPO pathways depend on intracellular iron metabolism, with disruptions leading to ferroptosis. The pathways and regulatory mechanisms of ferroptosis are outlined. (Figure 1). Specific types of cell ferroptosis play a crucial role in AS co-depression diseases.

## 5 Multiple cell ferroptosis promotes atherosclerosis

### 5.1 Ferroptosis of vascular endothelial cells promotes atherosclerosis

AS begins with vascular endothelial dysfunction, characterized by increased permeability, leukocyte adhesion, heightened inflammation, and accelerated plaque and thrombus formation. The negative effects of ferroptosis in endothelial cells during AS are well established. Bai et al. demonstrated that Ox-LDL, a potent pro-atherosclerotic factor, induces ferroptosis in mouse aortic endothelial cells by increasing iron deposition, lipid peroxides, and ROS while decreasing GPX4 levels. High-fat-fed ApoE^−/−^ mice exhibit larger atherosclerotic plaques, severe ferroptosis, increased NOX production, and reduced GSH and xCT expression in the thoracic aorta compared to wild-type mice (Bai et al., 2020). Li et al. showed that injecting endothelial progenitor cell-derived extracellular vesicles containing miR-199a-3p into high-fat fed APOE mice reduced iron accumulation and LPO, increased xCT, GSH, and GPX4 levels in the aorta, and resulted in smaller atherosclerotic plaques (Li et al., 2021). Peng et al. identified a positive correlation between endothelial cell function and N-acetylneuraminic acid (Neu5Ac) from glucose metabolism. Neu5Ac facilitates SLC3A2 binding to ubiquitin, initiating P62-mediated degradation, which leads to LPO and ferroptosis in high-fat-fed ApoE^−/−^ mice. This process is inhibited by Fer-1 (Xiang et al., 2023).

Iron accumulation from various sources significantly contributes to AS and vascular endothelial cell (VEC) ferroptosis. A key PL oxidation product present in atherosclerotic lesions is 1-palmitoyl-2-glutaryl-sn-glycero-3-phosphocholine (PGPC). Chen et al. show that PGPC treatment of human umbilical vein endothelial cells (HUVECs) increases fatty acid binding protein-3 (FABP3), ROS, LPO, and ferrous iron levels. It disrupts mitochondrial membrane potential and reduces glutathione and GPX4 expression. Ferroportin-1, a ferroptosis inhibitor, countered these effects. PGPC triggers ferroptosis through the CD36 receptor, impairing endothelial function (Chen et al., 2024). Iron accumulation from ferritin autophagy worsens AS, damaging endothelial cells. HUVEC exposed to high doses of ionizing radiation exhibits increased ferritin autophagy through the p38/NCOA4 pathway, leading to elevated iron levels, increased lipid peroxidation, and decreased antioxidant protein expression (GPX4, Nrf2, xCT, and SLC3A2). This cascade in significant VECs injury and ferroptosis (Wu Z. et al., 2023).

Additionally, enhancing VEC antioxidant activity is crucial for preventing ferroptosis and improving AS. Tumor necrosis factor-related protein 13 (CTRP13) is crucial for antioxidant defense and endothelial function. Du et al. developed a model of Ox-LDL-induced ferroptosis in HUVECs, showing that CTRP13 intervention increased antioxidant enzyme expression, preventing AS and protecting endothelial cells from ferroptosis (Du J. et al., 2024). Gualou-Xiebai (GLXB) inhibits ferroptosis in high-fat-fed ApoE^−/−^ mice by reducing mitochondrial damage, boosting glutathione and superoxide dismutase levels, and decreasing LPO and MDA levels. *In vitro*, GLXB mitigated erastin-induced ferroptosis, alleviating Ox-LDL-induced damage in HUVEC (Zhu L. et al., 2024). Qixian granule (QXG) is used to treat postmenopausal AS in women. Zhang et al. demonstrate that QXG inhibits GPX4 and FTH1-mediated ferroptosis by activating TRPML1 directly or through the GPER pathway, slowing AS progression. *In vitro*, QXG-treated serum prevents human aortic endothelial cells from proliferating, migrating, and inducing ROS and mitochondrial damage from Ox-LDL (Zhang et al., 2024).

### 5.2 Ferroptosis of macrophage promotes atherosclerosis

Macrophage death exhibits a dual role in atherogenesis. During the early stages of AS, moderate macrophage death reduces inflammation and metalloproteinase secretion. Conversely, in advanced stages, uncontrolled macrophage death exacerbates inflammation, promotes the formation of lipid-rich necrotic cores, and destabilizes plaques (Susser and Rayner, 2022; Wang L. et al., 2023). Numerous studies show the role of macrophage ferroptosis in promoting AS. Interleukin-23p19 (IL-23p19) is associated with cardiovascular conditions, including AS. Lu et al. created a mouse model of cardiac remodeling using transverse aortic constriction (TAC) and observed increased IL-23p19 expression levels in the heart after surgery, probably from cardiac macrophages. Silencing IL-23p19 reduces ferroptosis and M1 macrophage polarization in mice with TAC, improving cardiac remodeling and function recovery (Lu et al., 2024). Furthermore, Luo et al. report that activating Nrf2 and enhancing its nuclear translocation can inhibit Ox-LDL-induced ferroptosis in macrophages and resist ferroptosis in ApoE^−/−^ animals on a high-fat diet (Luo et al., 2024).

Erythrocyte phagocytosis and macrophage ferroptosis are essential triggers of AS. Advanced atherosclerotic plaques exhibit intra-plaque (IP) angiogenesis. The fragile and leaky nature of IP arteries releases erythrocytes, which are subsequently phagocytosed by macrophages. This process leads to increased intracellular iron levels, LPO, and apoptosis. *In vitro* studies show that erythrophagocytosis by macrophages induces atypical ferroptosis, potentially causing plaque instability. Elevated heme oxygenase 1 and ferritin levels observed during this process were reduced by the ferroptosis inhibitor UAMC-3203. Research by Pauline Prall and colleagues using ApoE^−/−^ mice reveals a significant decrease in carotid plaque thickness after 20 weeks, especially in plaques with hemorrhage or IP angiogenesis. This was accompanied by reduced expression levels of ferritin and IP heme oxygenase 1. Therefore, erythrophagocytosis-induced ferroptosis contributes to the formation of larger atherosclerotic plaques, but this effect can be reversed by UAMC-3203 (Puylaert et al., 2023). Liu et al. developed a red-line Jak2VF-expressing hyperlipidemic erythropoietin receptor Cre mice. They observed that these mice exhibit elevated iron and 4-HNE levels, increased erythrocyte phagocytosis by plaque macrophages, and enhanced macrophage ferroptosis, leading to greater necrosis in atherosclerotic plaques. These effects were amplified by selective erythropoiesis activation and inhibited by the ferroptosis inhibitor liproxstatin-1, indicating that Jak2VF signaling accelerates AS by promoting macrophage ferroptosis and erythrocyte phagocytosis (Liu W. et al., 2022).

### 5.3 Ferroptosis of vascular smooth muscle cells promotes atherosclerosis

Vascular smooth muscle cells (VSMCs) are essential in the early and advanced stages of AS. They infiltrate lesions, enlarge them and form a fibrous cap over the necrotic core. VSMC death causes vascular inflammation, loss of extracellular matrix (ECM) and collagen, weakening of the fibrous cap, and ultimately plaque rupture (Grootaert and Bennett, 2021; Xu X. D. et al., 2022). Ferroptosis of VSMCs is also a significant part of AS. Xie et al. observed that Fer-1 affects TFR1, FTH, and FTL expression in VSMC, reducing iron accumulation in atherosclerotic lesions *in vivo* and *in vitro*. Evidence suggests that VSMC ferroptosis may help enhance atherosclerotic lesions. Fer-1 fails to enhance the p53/SLC7A11/GPX4 pathway but improves the Nrf2/ferroptosis inhibitory protein 1 pathway, boosting resistance to LPO (You et al., 2023). Zhang et al. discovered that echinacea upregulates glutamate-cysteine ligase catalytic (GCLC) and modifier (GCLM) subunits in VSMC, maintaining the GSH balance essential for preventing AS. This regulation also reduces ferroptosis and matrix remodeling, both contributors to AS, by regulating GSH production (Zhang et al., 2023c). Mucosa-associated lymphoid tissue lymphoma translocation protein 1—a human cysteoaspartase paraprotein—induces ferroptosis in VSMCs through protein hydrolysis. A ferroptosis inhibitor reduces carotid artery neointimal plaques and AS in C57BL/6J and ApoE^−/−^ mice, indicating that suppressing ferroptosis in VSMCs may alleviate proliferative vascular disease (Yan et al., 2023).

### 5.4 Ferroptosis of foam cells promotes atherosclerosis

Foam cells are essential in developing atherosclerotic lesions, from initial stages to advanced plaques. Macrophages—the primary source of foam cells—proliferate in the arterial intima after pro-inflammatory activation by endothelial cells, allowing them to migrate across the endothelial barrier. A small portion of VSMC and endothelial cells can differentiate into macrophages, which may then become foam cells by engulfing lipids. VSMC can also develop into foam cells (Chistiakov et al., 2017; Maguire et al., 2019). During plaque formation, foam cell death—primarily from ferroptosis and autophagy—accelerates atherosclerotic plaque development. Insufficient autophagy inhibits Nrf2-mediated antioxidant defenses, promoting iron deposition and LPO, leading to ferroptosis (Peng et al., 2022). Li et al. used bioinformatics to explore the roles of ferroptosis and isocitrate dehydrogenase 1 (IDH1) in foam cell formation. They observed that ferritin-1 can reverse the increase in macrophage ferroptosis and IDH1 levels caused by Ox-LDL. Inhibiting IDH1 reduces damage and apoptosis in Ox-LDL-treated macrophages while increasing Nrf2 levels, thereby decreasing foam cell formation (Li B. et al., 2022). Su et al. treated THP-1 macrophages with ferric ammonium citrate (FAC) and Ox-LDL, using SRT1 to activate SIRT1720 and rapamycin along with chloroquine to modulate autophagy. They observed that FAC exposure increases lipid ROS levels, decreases GPX4 and SIRT1 expression, and elevates IL-1β and IL-18 levels while reducing foam cell activity compared to that of macrophage activity. These findings indicate that inhibiting ferroptosis in foam cells is essential for treating AS (Su et al., 2021).

In summary, ferroptosis in VECs, macrophages, VSMC, and foam cells contributes to AS. Treatment reduces ferroptosis levels in these cells and improves the condition, highlighting the importance of anti-ferroptosis strategies in the disease.

## 6 Multiple cells ferroptosis promotes depression

### 6.1 Ferroptosis of neurons promotes atherosclerosis

Brain cells are mainly involved in neurons, microglia, and astrocytes. Factors such as neuronal damage in the brain, inflammatory responses, oxidative stress, cell death, and altered neuroplasticity can influence depression (Malhi and Mann, 2018). Neurons are the fundamental structural and functional units of the nervous system. Studies show that iron-sag markers can be traced in cellular and animal models of neurodegenerative diseases (Cozzi et al., 2019). Fer-1 mitigates abnormal tsRNA expression profiles in the hippocampus tissues of the chronic unpredictable mild stress (CUMS) mouse model. Additionally, in an *in vitro* assay, ferroptosis is associated with reduced expression of SLC7A11 and GPX4 proteins, as well as ROS accumulation in corticosterone-constructed hippocampal progenitor neurons (Li E. et al., 2023). Xu et al. observed that alcohol consumption causes neuronal injury in the hippocampal and prefrontal cortex of mice. The alcohol-treated group shows reduced levels of synapse-associated proteins. Alcohol increases the number of iron-positively stained cells and the expression of the TFR1 protein. Following iron staining, GPX4 expression is minimized in the alcohol group. Conversely, the ferroptosis inhibitor ferritin-1 significantly prevents alcohol-induced damage to neurons *in vitro* and reverses the expression of the proteins mentioned above (Xu C. et al., 2022). In CUMS animals, decreased ferritin light chain 1 and Brain-derived neurotrophic factor (BDNF) levels are observed in the proteomics of hippocampal neurons, which are associated with hippocampal neuronal survival. Additionally, ferroptosis-associated proteins are differently expressed in these animals (Cao et al., 2021). Li et al. further demonstrated that enhancing hippocampal neuronal BDNF levels could prevent hippocampus neuronal ferroptosis and potentially enhance the antidepressant effects of electroconvulsive therapy (Li X. et al., 2023). The studies mentioned above highlight the significant roles that neuronal loss and ferroptosis play in depression.

### 6.2 Ferroptosis of microglia promotes atherosclerosis

Microglia make up approximately 0.5%–16.6% of the cells in the human central nervous system and are the sole remaining mononuclear phagocytes within the brain parenchyma (Askew et al., 2017). Microglia plays various roles in brain development, synaptic plasticity, neuronal synchronization, and neuroimmune homeostasis from embryo development to adulthood. They also interact with neurons, astrocytes, and oligodendrocytes via chemical signals or direct contact throughout the life cycle of an organism. Neuroinflammation progresses because of the combined effects of inflammation and microglial iron buildup, which can be inhibited to stop the progression of neuroinflammation (Liu S. et al., 2022). Depression is frequently accompanied by inflammation, and microglia are essential starting materials and controllers of the cynaroside (CNS) inflammatory cascade responses (Wang H. et al., 2022). Neuroinflammation is another significant mechanism in many neurodegenerative diseases and is often associated with persistent activation of microglia. Microglial energy metabolism is gaining prominence in neurodegenerative disorders, as numerous brain diseases are associated with changes in brain energy metabolism and constant inflammation. Moreover, energy metabolism strongly influences the inflammatory response of microglia (Aldana, 2019).

Inhibiting microglia ferroptosis to ameliorate CNS inflammation is a promising therapeutic tool for depression. Wang et al. utilized shRNA silencing of the microglial GPX4 gene to assess the anti-inflammatory effects of saikosaponin B2 (SSB2) in LPS-induced primary microglia and CUMS-induced mouse models of depression. Consequently, SSB2 exhibits anti-ferroptosis and anti-neuroinflammatory effects, attenuating the activation of the LPS-induced primary microglial TLR4/NF-κB signaling pathway in a GPX4-dependent manner (Wang et al., 2023c). Jiao et al. suggest that the antidepressant mechanism of Prowessan may involve the enhancement of the iron-death-associated PEBP1-GPX4 pathway. This pathway can control the expression of PEBP1, ERK1/2, GPX4, FTH1, ACSL4, and COX2, thereby enhancing the function of hippocampal astrocytes and microglial cells in CUMS model mice (Jiao et al., 2021). Yang et al. investigated the role of gallic acid in preventing ferroptosis in spinal microglial cells in rats with chronic pain and depression. They discovered that the anti-inflammatory and antioxidant properties of gallic acid decrease tissue iron concentration, improve mitochondrial damage, inhibit the P2X7-ROS signaling pathway, and reverse behavioral changes in these rats (Yang et al., 2023) (Table 1).

**TABLE 1:** The effects of microglia ferroptosis on depression.

Interventions	Subject	Pathway/Protein	Mechanism	Results	Reference
Saikosaponin B2	CUMS mice	Inhibition of TLR4/NF-κB, GPX4↑	Anti-inflammatory, antioxidant, anti-ferroptosis	Depression↓	Wang et al. (2023c)
XiaoYao San	CUMS mice	Activation of PEBP1-GPX4	Improve microglia cell function	Depression↓	Jiao et al. (2021)
Gallic acid	CUMS rat	Adjustment P2X7-ROS	Inhibit ferroptosis in spinal microglia	Pain↓ and Depression↓	Yang et al. (2023)
Eicosapentaenoic acid	Mice	Nrf2↑ NLRP3↓	Inhibit microglia M1 polarisation and ferroptosis	Seizures↓ and Depression↓	Wang et al. (2022b)
Acupuncture	CUMS rat	SIRT1↑ Nrf2↑HO-1↑ GPX4↑	Anti-inflammatory and antioxidant, inhibits activation of microglia	Depression↓	Shen et al. (2024)
LPS	BV-2 cells	ALKBH5-PRMT2-β-catenin-GPX4	Promoting microglia ferroptosis and polarisation	Depression↑	Mao et al. (2024)
Cynaroside	CUMS mice	IRF1/SLC7A11/GPX4↑	Inhibition of microglia polarisation to M1 phenotype and reduction of inflammation and ferroptosis	Depression↓	Zhou et al. (2024)
Hydrogen sulfide	CUMS mice/BV-2 cells	SLC7A11/GPX4/CBS↑	Anti-inflammatory, Fe^2+^↓ MDA↓ ROS↓ lipid peroxide↓	Depression↓	Wang et al. (2021b)

CUMS: Chronic unpredictable mild stress; TLR4:Toll-like receptor 4; NF-κB:Nuclear factor kappa-B; GPX4:Glutathione Peroxidase 4; PEBP1: Phosphatidylethanolamine Binding Protein 1; ROS:Reactive oxygen species; Nrf2:Nuclear factor ervthroid2-related factor 2; NLRP3:NOD-like receptor family pyrin domain containing 3; SIRT1:Sirtuin 1; HO-1:Heme oxygenase 1; ALKBH5:Alk B homolog 5; IRF1:Interferon Regulatory Factor 1; SLC7A11: Solute carrier family 7 member 11; MDA: malondialdehyde.

### 6.3 Ferroptosis of astrocytes promotes atherosclerosis

Astrocytes—the most abundant and largest class of glial cells in the mammalian brain—maintain and divide neuronal cells, aid in creating the blood-brain barrier, and regulate the onset of several disease processes (Khakh and Sofroniew, 2015). In culture, astrocytes are observed to shield neurons from oxidants and excitotoxins. This neuroprotective effect is thought to result from their ability to absorb glutamate and recycle free radicals. Astrocytes are involved in numerous neurodegenerative disorders and can significantly influence depression. In Orai1 knockout mice, the LPS-induced depression-like behavior, including the helplessness and pleasure deficit, is improved in astrocytes (Novakovic et al., 2023). Mice subjected to 6 weeks of prolonged stress followed by 6 weeks of social isolation exhibit symptoms of depression, accompanied by elevated levels of astrocyte activation in the dorsal and ventral dentate gyrus regions of the hippocampus (Du Preez et al., 2021). Prenatal exposure to air pollution in rodents causes astrocyte degeneration, microglia activation, and disruption of the blood-brain barrier, leading to depressive tendencies in rats. These rats also show a twofold increase in hippocampus iron accumulation (Woodward et al., 2018).

Inhibition of astrocyte activation and ferroptosis ameliorates depression. CUMS depression model mice treated with the compound Chinese medicine XiaoYao San for 2 weeks showed a decrease in hippocampal body weight, total iron, and ferrous iron content. Changes were made in the concentrations of proteins associated with ferroptosis, including GPX4, FTH1, ACSL4, and COX2. The expression of glial fibrillary acidic protein—a marker of activated astrocytes—decreases, enhancing astrocyte glial cell activity (Jiao et al., 2021). Diffuse depression in neurons may be caused by a discrepancy in the antioxidant system, where the energy metabolic state of astrocytes plays a crucial role. ATP depletion in astrocytes leads to reduced glutathione levels and increased ferrous ions, reducing their antioxidant capacity. The iron chelating agent deferoxamine reduces astrocyte damage, suggesting that improvements in astrocyte functionality are partly attributed to the inhibition of their ferroptosis (Lian and Stringer, 2004).

**FIGURE 2:**
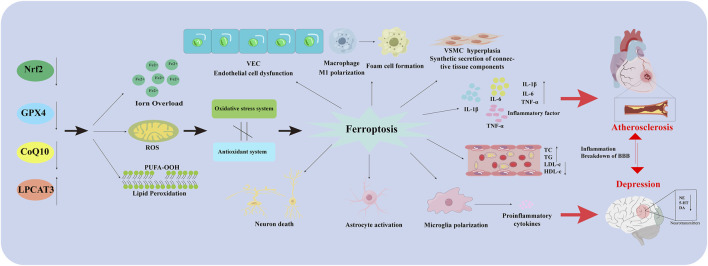
Ferroptosis in different types of cells in AS co-depression disease. Nrf2: Nuclear factor erythroid 2-related factor 2; GPX4: Glutathione peroxidase 4; CoQ10H2: Reduced coenzyme Q10; LPCAT3: Lysophosphatidylcholine acyltransferase 3; VEC: Vascular endothelial cell; PUFA-OOH: Polyunsaturated fatty acid-OOH; ROS: Reactive oxygen species; VSMC: Vascular Smooth Muscle Cell; IL-1β: Interleukin-1 beta; IL-6: Interleukin- 6; TNF-α: Tumor necrosis factor-α; TC: Total Cholesterol; TG: Triglyceride; LDL-C: Low-Density Lipoprotein Cholesterol; HDL-C: High density lipoprotein cholesterol; BBB: Blood Brain Barrier; NE: Norepinephrine; 5-HT: 5-hydroxy tryptamine; DA: Dopamine.

As mentioned above, microglia, neurons, and astrocytes are involved in the pathological depression process. Changes in indicators of cellular ferroptosis are observed in all three types of cells, and effective improvements can be made using drugs or other intervening behaviors. This suggests that modulating ferroptosis in these 3 cell types plays an important role in depression (Figure 2).

## 7 Ferroptosis and cardiovascular disease co-depression

We have previously discussed the separate roles of iron death in AS and depression. Ferroptosis also plays a significant role in comorbidity. After a closed head injury, ApoE^−/−^ mice exhibit elevated levels of iron in their brains, suggesting that ApoE^−/−^ deficiency may increase the risk of oxidative damage to hippocampal tissue (Guaraldi and Shea, 2018). A randomized controlled clinical study of 26 patients with myocardial infarction, anxiety, and depression, compared to that of 26 healthy individuals, revealed that ferroptosis is involved in the pathogenesis of cardiovascular and cerebrovascular diseases associated with depression. *In vitro* studies show that upregulating sestrin2 decreases type I/II collagen and KEAP1 mRNA expression while increasing GPX4 and Nrf2 mRNA levels. Similar findings were observed with sestrin2 downregulation (Qian et al., 2023). Ferritin improves cognition by decreasing ROS and glutathione depletion in the MCAO model and downregulating elevated ferroptosis factors P11 and SLC53A7 (Ko et al., 2023).

## 8 Ferroptosis affects atherosclerosis and depression through different mechanisms

We have summarized the key mechanisms of cellular ferroptosis in AS and depression. However, disease development is often complex, involving multiple mechanisms and their interactions. Ferroptosis is also closely associated with inflammation, mitochondrial dysfunction, and the gut microbiota, which also play important roles in AS and depression.

### 8.1 Ferroptosis exacerbates peripheral inflammation

AS—a chronic inflammatory vascular disease—is closely associated with inflammation in its development (Kong et al., 2022). Macrophage-mediated inflammatory responses significantly contribute to AS progression, with various macrophage types observed in plaques. Excessive lipid uptake primarily drives polarization into M1-type macrophages. M2-type macrophages contain less intracytoplasmic iron and metabolize it more quickly than that of M1-type macrophages, which accumulate iron owing to their high ferritin content. Iron levels influence macrophage polarization: low levels inhibit anti-inflammatory responses, while high levels promote them (Cornelissen et al., 2019). Elevated iron levels increase macrophage secretion of matrix metalloproteinases, degrading the ECM and inducing AS plaque rupture. Iron overload also hinders lipoxygenase binding to the nuclear membrane, exacerbating inflammation and promoting AS formation (Ma et al., 2022). Ferroptosis—along with iron overload and imbalanced redox reactions—is closely associated with AS, contributing to macrophage foam cell formation. It increases ROS expression by promoting P53 acetylation, driving macrophage differentiation into the M1 phenotype (Zhou et al., 2018). Additionally, M2-type macrophages can be converted into M1-type macrophages when induced by iron nanoparticles (Laskar et al., 2013). Therefore, ferroptosis enhances M1-type macrophage polarization, worsens peripheral inflammation, and accelerates AS progression (Ma et al., 2022).

### 8.2 Ferroptosis exacerbates central inflammation

Neuroinflammation is primarily initiated by astrocytes and microglia in response to factors such as injury, infection, exposure to toxins, or autoimmune reactions. Microglia—the resident immune cells of the CNS—are characterized by elevated levels of iron (Liu S. et al., 2022). This is closely associated with iron overload-mediated depression and abnormal glial cell activation (Zeng et al., 2023). Research shows that synaptic plasticity theory is a widely accepted mechanism significant to the pathophysiology of depression, with BDNF signaling being one of these. BDNF downregulation, driven by various factors, causes neurotoxic effects and is crucial for synaptic plasticity in depression (Li Y. et al., 2024; Lomeli et al., 2024). Li et al. observed that iron overload through the ferricyanide/BDNF pathway may damage hippocampal neurons by reducing BDNF levels (Li J. et al., 2024). They also suggest that etomidate may have antidepressant effects by increasing BDNF, thereby protecting hippocampal neurons from ferroptosis (Li X. et al., 2023). Additionally, lactoferrin—an iron-binding glycoprotein—reduces NF-κB (p65) and TNF-α levels, lessening inflammation and depressive behaviors in rats subjected to chronic restraint stress. This is supported by findings in CUMS model mice, where iron buildup in hippocampal microglia influences neuronal degeneration and death (Ahmed et al., 2023; Gao et al., 2019). Microglia activation, increased pro-inflammatory cytokine expression, and ferroptosis in the hippocampus of CUMS mice are reversible with further erastin therapy (Ma et al., 2024). Zhang et al. observed that deferoxamine reverses neuropathological changes caused by inflammatory factor upregulation in a CUMS mouse model (Zhang W. et al., 2022). These findings indicate a clear link between depression onset and neurotoxicity from excess iron.

### 8.3 Ferroptosis affects atherosclerosis by modulating mitochondrial function

Iron is essential for lipid metabolism, protein synthesis, cellular respiration, and DNA synthesis, with mitochondria playing a key role in iron metabolism. Iron also contributes to adaptive mechanisms in cardiac control (Ravingerová et al., 2020), but its regulatory role in iron metabolism is often overlooked. The heart derives energy from iron-sulfur clusters (ISCs), produced by mitochondria, the only sites of hemoglobin production. These ISCs contain hemoglobin and catalyze electron transfer through the bidirectional oxidation states of iron. Excessive iron accumulation in mitochondria causes oxidative stress, generating harmful free radicals that damage DNA, proteins, and lipids, ultimately impairing cardiac function (Kumfu et al., 2012). Altered mitochondrial morphology distinguishes ferroptosis from other cell death types. This alteration can lead to excessive ROS production, damaging VECs and causing mitochondrial dysfunction. Aortic endothelial cells (MAECs) from high-fat-fed ApoE^−/−^ mice exhibited increased expression of mitoferrin 2 (Mfrn2), an iron transporter in the inner mitochondrial membrane. Silencing the Mfrn2 gene prevents mitochondrial iron overload in MAECs (Wang D. et al., 2021). Research on the role of ferroptosis in mitochondrial function and its effects on AS is limited. However, the studies mentioned above highlight its significance in AS and offer new insights for investigating the pathological mechanisms of AS through ferroptosis.

### 8.4 Ferroptosis affects depression by modulating mitochondrial function

Mitochondria are essential for cellular energy metabolism and play a key role in inducing ferroptosis through various metabolic processes. Intracellular ROS are primarily produced by mitochondrial metabolism. Superoxide is produced when electrons leak from, ETC., complexes I and III and they are converted into hydrogen peroxide by superoxide dismutase. This hydrogen peroxide reacts with ferrous ions to form hydroxyl radicals, which then transform the bis-allyl hydrogens in PUFAs into PUFA radicals. In the presence of oxygen, these unstable free radicals quickly form PUFA peroxyl radicals, which ultimately convert into PUFA hydroperoxides. This process promotes LPO, leading to ferroptosis owing to ROS produced by mitochondria. Elevated ferrous ions and iron homeostasis imbalances can further increase ROS through the Fenton reaction, also contributing to ferroptosis. Numerous researches show that oxidative stress and disruption of the electron transport chain in mitochondria are closely related to depression (Jiang et al., 2024; Khan et al., 2023; Scaini et al., 2022). Among them, mitochondria-mediated production is positively associated with depression incidence. Excess iron ions disrupt the electron transport chain and reduce ATP synthesis in the mitochondria. Research indicates that individuals with severe depression exhibit lower ATP and glucose metabolism in brain regions associated with mood regulation, including the bilateral insula, nucleus accumbens, and cingulate gyrus (Su et al., 2014). ATP levels in the hippocampus and prefrontal cortex of CUMS depression model mice were reduced, but injecting ATP into the lateral ventricle may significantly enhance their depressive behavior (Cao et al., 2013; Shen et al., 2020). Furthermore, issues with mitochondrial iron metabolism problems may lead to depression by disrupting mitochondrial structure and function, which are key sources of cellular ROS. This disruption may also relate to depression management via the ferroptosis signaling pathway (Song Y. et al., 2023).

### 8.5 Ferroptosis affects atherosclerosis by regulating gut flora

Gut microbes also play a key role in AS. Studies show significant changes in the gut microbiota of individuals with ASCVD. In a metagenomic analysis of the gut microbiome of 218 patients with ASCVD and 187 healthy patients, Jie et al. identified a strong association between gut microbiota and ASCVD (Jie et al., 2017). Animal models indicate that gut microbiota may contribute to ASCVD through mechanisms such as direct host invasion, immune system activation through lipopolysaccharide, altered metabolism, and the release of metabolites into the bloodstream. Trimethylamine N-oxide is the most established link to ASCVD, supported by human and animal studies (Barrington and Lusis, 2017). Recent studies show that iron storage in mice correlates positively with *Clostridium* spp. iron utilization, which is enhanced by dietary iron, intravenous iron, and chronic blood transfusions (La Carpia et al., 2019). An increase in *Clostridium* species has been observed in AS (Konturek et al., 2015). Additionally, a randomized controlled trial reveal that Qing-Xin-Jie-Yu Granule can modify gut flora, reduce AS, and prevent ferroptosis by modulating the GPX4/xCT pathway, thereby stabilizing AS plaques (Zhang et al., 2023b). These findings highlight the significant function of ferroptosis in AS and suggest that gut microbiota are a major contributor.

### 8.6 Ferroptosis affects depression by regulating gut flora

**FIGURE 3:**
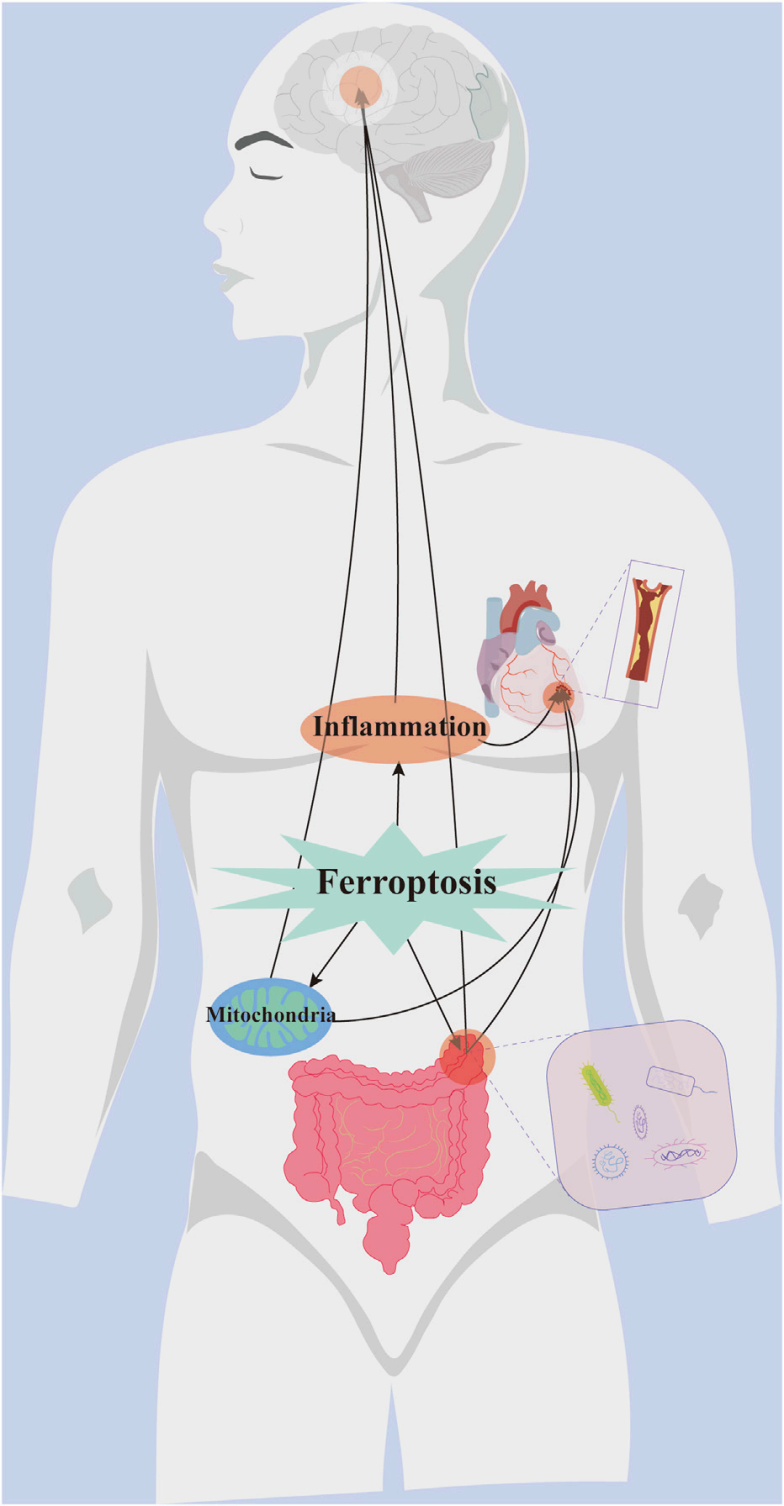
Crosstalk between ferroptosis and common pathogenesis of AS co-depression disease.

The brain-gut axis is a bidirectional communication network that connects cognitive and emotional functions of the brain to gut activity through neural pathways, neuroendocrine and neuroimmune systems, and gut microbiota (Mayer et al., 2022). Disturbances in gut microbiota and its metabolites have been observed in individuals with depression and animal models, suggesting a link between depression and disrupted gut microbiota, as well as impaired neural communication (Liu L. et al., 2023; Wang Y. et al., 2024). Imbalances in serum iron homeostasis can trigger inflammation and gut microbial diseases, leading to brain stress responses. Increased IL-1β levels elevate iron regulatory protein 1 and TFR1 in oligodendrocytes, while reduced iron transporter protein 1 expression leads to intracellular iron accumulation, contributing to neurodegenerative diseases (Yang J. et al., 2024). Studies indicate a strong connection between depression onset and IL-1β-mediated signaling pathways (García-García et al., 2022; Ng et al., 2022; Wang et al., 2018), with IL-6 playing a similar role (Zhang G. et al., 2023). Individuals with depression exhibit reduced BDNF levels, highlighting its potential as a diagnostic biomarker (Rana et al., 2021). Ferroptosis is linked to depression by suppressing BDNF expression, while gut microorganisms influence BDNF and modulate depression through the brain-gut axis (Matin and Dadkhah, 2024). Depression may be associated with gut flora dysbiosis, and probiotics could help alleviate its symptoms. Iron metabolism and ferroptosis may induce neurological damage through interactions with the central nervous system, gut microbiota, and brain-gut axis, positioning them as potential therapeutic targets for depression (Figure 3).

### 8.7 The role of ferroptosis in atherosclerosis with depression

Ferroptosis influences the onset of AS and depression through inflammation, mitochondrial dysfunction, and gut microbiota, respectively. It also plays an important role in the comorbidity of atherosclerotic cardiovascular disease and depression. In patients with chronic stable angina pectoris complicated by depression and anxiety, Melissa officinalis supplements have shown significant improvement in symptoms. Melissa officinalis exhibits strong antioxidant properties, regulates lipid metabolism (Petrisor et al., 2022), and can also inhibit ferroptosis (El Hajj et al., 2023). In patients with AS and depression, elevated levels of the inflammatory factors IL-6 and IL-10 are observed (Mousa et al., 2022), while ferroptosis exerts a significant pro-inflammatory effect. In ApoE^−/−^ mice subjected to chronic mild stress, lipid metabolism disorders, increased adipocyte hypertrophy, and reduced PPARG gene expression promote AS, while PPARG exhibits an anti-ferroptosis effect (Mao et al., 2023). Therefore, the role of ferroptosis in AS and depression is bidirectional, involving interactions through various disease mechanisms.

In summary, the significant role of ferroptosis in AS and depression has been highlighted. Ferroptosis contributes to the onset and progression of AS co-depression directly or by influencing other mechanisms. Targeted regulation of ferroptosis through drugs could represent a novel approach for treating AS-depression comorbidities. However, commonly used ferroptosis modulators are often associated with significant side effects, including pulmonary toxicity (Parry et al., 2002), liver damage, and neurotoxic effects (Zaafar et al., 2024). Currently, Chinese medicine offers promise for treating AS and depression. Its compounds exhibit anti-ferroptosis effects with minimal side effects, enhancing patient acceptability. Therefore, we will review the anti-ferroptosis effects of Chinese medicine compounds and monomers in AS and depression.

## 9 The development of ferroptosis-focused Chinese medicine for the treatment of depression and atherosclerosis

### 9.1 Treatment of atherosclerosis with a Chinese medicine monomer targeting ferroptosis

Paeonia lactiflora, a medicinal herb native to China, is the source of Paeonol (Pae), a bioactive compound with potent antioxidant and lipid-lowering properties (Shi et al., 2020). Studies indicate that Pae reduces lipid accumulation and foam cell growth, thereby lowering atherosclerotic plaque formation (Shi et al., 2022). Additionally, studies reveal that Pae enhances Nrf2 protein expression, potentially preventing lipid accumulation in foam cells by reducing ferroptosis. Gao et al. reveal that Pae effectively reduced fat growth and aortic ferroptosis in ApoE^−/−^ mice, outperforming ferritin-1 and simvastatin. *In vitro*, Pae inhibited lipid accumulation from Ox-LDL in foam cells undergoing ferroptosis. The SIRT1/Nrf2/GPX4 pathway was identified as a protective mechanism, with SIRT1 knockdown reversing this effect. Therefore, Pae may inhibit lipid accumulation and alleviate AS by targeting this pathway (Gao et al., 2024).

Hydroxysafflor yellow A (HSYA), a monochalcone glycoside derived from the Asteraceae family, is used to manage and prevent ischemic cerebrovascular diseases. Its anti-inflammatory, antioxidant, and anti-angiogenic properties help alleviate AS (Feng et al., 2023; Xue et al., 2021). HSYA reduces oxidative damage to HUVECs caused by H2O2 and inhibits ferroptosis during reperfusion, suggesting a protective role in heart tissues. Rong et al. administered HSYA to ApoE^−/−^ mice and applied it in an Ox-LDL-induced hyperlipidemic model in HUVEC. HSYA suppressed ferroptosis in HUVEC, increased GSH, SLC7A11, and GPX4 levels, and significantly reduced atherosclerotic plaque formation. It also downregulated miR-429, which regulated SLC7A11 expression. After SLC7A11 siRNA transfection or miR-429 mimicry, the antioxidative stress and anti-ferroptosis effects of HSYA were reduced. Therefore, HSYA holds promise as a therapeutic agent for AS (Rong et al., 2023).

Echinatin, a chalcone isolated from the Chinese herb licorice, possesses anti-inflammatory and antioxidant properties. Zhang established a mechanical property-based screening approach to identify compounds that alleviate arterial wall stiffness by affecting the interactions between VSMCs and the ECM. The study reveals that echinatin reduced ECM stiffness surrounding cultured VSMCs and upregulated glutamate cysteine ligases (GCLs) expression, including the catalytic (GCLC) and regulatory (GCLM) subunits. Further studies show that the upregulation of GCLC/GCLM in VSMC by echinatin helps maintain the homeostasis of GSH metabolism. Adequate glutathione is essential for counteracting AS (Zhang et al., 2023c). Icariin, the main active ingredient of epimedium, improves endothelial cell dysfunction. Wang et al. investigated the protective effect of icariin on Ox-LDL-treated VECs and ApoE^−/−^ mice fed a high-fat diet. The findings reveal that icariin reduces the atherosclerotic plaque area and collagen fibers in aortic sinus tissue while enhancing mitochondrial activity and membrane potential. The levels of ROS in VECs were decreased. *In vivo* experiments, icariin reduced ferroptosis alleviated atherosclerotic lesions, and increased TFEB nucleation rate. These findings suggest that icariin could be a potential candidate for preventing VEC ferroptosis in cardiovascular disease treatment (Wang et al., 2023d). Tanshinone IIA (TSA) protects endothelial tissue from injury. We investigated its effect on ferroptosis in human coronary endothelial cells treated with TSA. TSA significantly reduces the excessive accumulation of total cell ROS and lipid ROS induced by ferroptosis inducers, restores glutathione content, and promotes nuclear translocation of Nrf2 (He et al., 2021).

### 9.2 Treatment of as with a Chinese medicine compound targeting ferroptosis

TCM compounds are widely used in AS treatment due to their multi-target effects, including the anti-ferroptosis mechanism. MaiJiTong Granules (MJT), a traditional Chinese medicine (TCM) formulation, contains Mucuna pruriens, Astragalus, Cinnamon, Danshen, Poria, and Paeonia lactiflora, which effectively treat cardiovascular diseases. Key ingredients, such as Astragalus saponin IV and ferulic acid, inhibit ferroptosis (Peng et al., 2021). Jia et al. developed a mouse model of AS through high-fat feeding to assess the efficacy of MJT in preventing and treating ferroptosis and AS. The study reveals that MJT exerts anti-inflammatory effects, lowers LDL levels, inhibits foam cell formation, and reduces plaque area and instability, thereby slowing AS progression. Additionally, MJT promotes ferroptosis and alleviates iron dysregulation and LPO during atherogenesis, reducing DMT1 and SOCS1/p53 expression through STAT1 phosphorylation (Shi et al., 2024).

DiDang decoction (DDD) is a TCM formula comprising rhubarb, peach kernel, leech, and gadfly, known for its ability to break up silt. Documented in the Typhoid Fever and Golden Chamber, it is used in China to treat AS and hyperlipidemia. DDD mitigates AS by preventing vascular fibrosis, preserving endothelial function, and reducing plaque rupture (Ren et al., 2017; Zhou et al., 2020). Wu et al. investigated the anti-ferroptosis effects of DDD through network pharmacology and *in vitro* experiments. They found that DDD therapeutically targets AS by enhancing mitochondrial function through the HIF-1 signaling pathway, reducing ROS levels, and modulating GPX4, Bcl2, and Bax protein levels, thereby benefiting AS and hyperlipidemia (Wu et al., 2023a).

The Qingxin Jieyu Granule (QXJYG), created by academician Chen Keji, is a key formula for ASCVD based on the “blood stasis and toxin” theory. It features ingredients such as Astragalus, Dangshen, and Huo Xiang. Previous studies show that QXJYG granules reduce inflammatory factors, modify intestinal flora to alleviate AS, and regulate iron metabolism in macrophages, stabilizing atherosclerotic plaques in individuals with stable coronary artery disease. Zhang et al. used RSL3 to induce macrophage ferroptosis *in vitro* and investigated the effects of Qing Xin Xie Du Granule on ferroptosis in ApoE^−/−^ mice. QXJYG reduced inflammatory factors associated with ferroptosis, lowered lipid peroxides such as MDA, enhanced antioxidant capacity, and inhibited AS progression and plaque vulnerability. Additionally, QXJYG decreased total iron content compared to that of the model group and significantly increased GPX4/xCT expression in aortic tissues, reversing RSL3-induced ferroptosis in macrophages (Zhang et al., 2023b) (Table 2).

**TABLE 2:** Traditional Chinese medicine targeting ferroptosis in the treatment of AS.

Interventions	Subject	Mechanism	Results	Reference
Paeonol	Foam cell	Upgrading of SIRT1/Nrf2/GPX4	Atherosclerosis↓	Gao et al. (2024)
Hydroxysafflor yellow A	HUVEC	Regulating miR-429/SLC7A11	Atherosclerosis ↓	Rong et al. (2023)
Echinatin	VSMC	Upgrading of GCLC/GCLM	Atherosclerosis ↓	Zhang et al. (2023c)
Icariin	VEC	Inhibits ROS and promotes autophagy	Atherosclerosis ↓	Wang et al. (2023d)
Tanshinone IIA	HCAEC	Inhibiting total ROS and lipid peroxidation, activation of Nrf2	Atherosclerosis ↓	He et al. (2021)
MaiJiTong granule	LDLR^−/−^ mice	Activation of STAT6, DMT1 and SOCS1/P53 signalling pathways	Atherosclerosis ↓	Shi et al. (2024)
DiDang decoction	L-O2 cell	Activation of HIF-1 signalling pathway, reduces ROS levels and inhibits ferroptosis	Atherosclerosis ↓ Hyperlipidemia ↓	Wu et al. (2023a)
Qing-Xin-Jie-Yu Granule	J744A.1 cells	regulating the GPX4/xCT signaling pathway	Atherosclerosis ↓	Zhang et al. (2023b)

HUVEC:human umbilical vein endothelial cells; VSMC:vascular smooth muscle cell; GCLC: Glutamate-Cysteine Ligase; GCLM: Glutamate-cysteine ligase modifying subunit; VEC: vascular endothelial cell; ApoE^−/−^:Apolipoprotein E Knockout; STAT6: Signal transducer and activator of transcription 6; DMT1:Divalent metal transporter 1; SOCS1: Suppressor of cytokine signaling 1; HIF-1: Hypoxia-inducible factor 1; LDLR^−/−^:Low-Density Lipoprotein Receptor Knockout.

### 9.3 Treatment of depression with a Chinese medicine monomer targeting ferroptosis

Quercetin, a flavonoid found in many fruits and vegetables, exhibits anti-inflammatory, antioxidant, and antidepressant effects. It also exerts various pharmacological effects, including modulation of the intestinal microbiota (Li B. et al., 2024), anti-apoptosis (Ge et al., 2024), inhibition of inflammatory vesicle activation (Du L. et al., 2024), and modulation of glutamate receptors (Wang M. et al., 2024). Quercetin regulates micronutrients by chelating iron, scavenging ROS, and reducing ferroptosis induced by various pathological factors (Zoidis et al., 2021). Wang et al. used a rat model of perimenopausal depression (OVX-CUMS) to examine the effects of quercetin on serum elemental changes. The findings reveal that OVX-CUMS rats exhibit increased iron levels in the blood and prefrontal cortex, accompanied by reduced expression of ferroptosis-associated proteins SLC7A11 and GPX4, which improve following quercetin treatment (Wang D. et al., 2024). Furthermore, Zhu et al. investigated the effects of quercetin on the lipid metabolism gene PTGS2 in breast cancer-related depression. They found that quercetin improves lipid metabolism and intestinal flora while reducing ferroptosis markers (total iron, Fe2+, MDA, and ROS). These findings confirm the potential of quercetin to inhibit neuronal ferroptosis and enhance the immune response, offering relief from depression-associated breast cancer (Zhu Q. et al., 2024).

Gastrodin, the main active compound in the Aspalathus rhizome, exhibits significant neurobiological effects. It reduces β-amyloid deposition, inhibits glutamate production, prevents ferroptosis, and restores synaptic plasticity. Furthermore, it increases BDNF levels, promotes neurogenesis, inhibits microglial activation, and regulates dopamine concentrations. Clinical studies show the effectiveness of gastrodin in managing post-stroke depression (Berezutsky et al., 2022). Catalpol, derived from dihuang, a cyclic enol ether terpene in TCM, exhibits hypoglycemic, depressive, and neuroprotective effects (Wang Y. L. et al., 2021). Wu et al. used a network pharmacology approach to predict that Catalpol could inhibit ferroptosis and mitigate fluoxetine-induced liver injury. Subsequent molecular docking reveals that ATF3/FSP1-mediated ferroptosis plays a major role in fluoxetine-induced hepatic injury. Additionally, Catalpol can reverse and enhance the antidepressant effects of fluoxetine (Wu X. et al., 2024). CNS, an antioxidant flavonoid from Chinese honeysuckle, has recently garnered attention for its potential antidepressant benefits (Yang et al., 2021). Zhou et al. conducted transcriptome analysis and validation, showing that CNS inhibits LPO, ferroptosis, and inflammatory polarization through the IRF1/SLC7A11/GPX4 signaling pathway. Furthermore, *in vivo* experiments showed that CNS exhibited therapeutic effects comparable to those of fluoxetine, effectively ameliorating symptoms of anxiety, despair, and anhedonia while also inhibiting microglial activation in the hippocampus of mice subjected to CUMS. CNS was found to mitigate inflammation, lower ferroptosis levels, prevent microglial polarization to the M1 phenotype and promote overall mental health (Zhou et al., 2024). Additionally, herbal extracts such as silybin (Liu P. et al., 2023) and Lycium barbarum glycopeptide (Dai et al., 2023) have been shown to target ferroptosis with antidepressant effects.

### 9.4 Treatment of depression with a Chinese medicine compound targeting ferroptosis

Chinese herbal compounds are increasingly used to target ferroptosis in depression treatment. The antidepressant properties of Pure Essence, documented in the prescription compendium of the Taiping Welfare Pharmacy Bureau during the Song Dynasty, have been thoroughly validated through TCM theory, clinical applications, and pharmacological research. Furthermore, it is considered one of the classical formulas for antidepressant treatment (Ran et al., 2024). Animal studies show that it ameliorates depressive-like behavior in CUMS rats, enhances neurotransmitter transmission, and modulates gene expression in astrocytes (Wu Y. et al., 2024). Recent studies have increasingly focused on the role of ferroptosis in the pathophysiology of depression. Jiao et al. showed that free-powder administration could alleviate depressive behaviors in CUMS mice by modulating PEBP1-GPX4-mediated ferroptosis in the hippocampus. This effect was evidenced by alterations in total and ferrous iron levels in the hippocampus and an increase in the expression of PEBP1, ERK1/2, and key ferroptosis-related proteins, including GPX4, FTH1, ACSL4, and COX2 (Jiao et al., 2021). Similarly, Di Huang Yin Zi, another Chinese herbal compound, has shown potential antidepressant effects in the brains of post-stroke depressed rats. These effects are associated with increased ROS and MDA levels, as well as modifications in ferroptosis-related markers, such as Fe^2+^, SLC7A11, and GPX4 (Yang et al., 2024c). In addition, a recent study suggests that Suanzaoren decoction modulates the DJ-1/Nrf2 signaling pathway, alleviating neuronal loss, synaptic damage, and ferroptosis associated with Alzheimer’s disease. This modulation is evidenced by the upregulation of FPN1, DJ-1, Nrf2, GPX4, and SLC7A11 proteins in the hippocampus, coupled with the downregulation of TfR1, FTH1, FTL, and ACSL4 proteins (Long et al., 2024). Suanzaoren Decoction also exhibits multi-pathway and multi-targeted antidepressant effects, including anti-inflammatory actions and gut flora regulation (Du et al., 2023; Du Y. et al., 2024). While studies on antidepressants have not specifically focused on ferroptosis, it is likely that the actions of Suanzaoren Decoction contribute to the modulation of ferroptosis in depression (Table 3).

**TABLE 3:** Traditional Chinese medicine targeting ferroptosis in the treatment of depression.

Interventions	Subject	Mechanism	Results	Reference
Quercetin	OVX-CUMS rat	Fe↓GPX4 and SLC7A11↑	Depression↓	Wang et al. (2024a)
Quercetin	BALB/c mice and primary hippocampal neurons	Fe, Fe^2+^,MDA and ROS↓	Depression↓	Zhu et al. (2024b)
Gastrodin	Mice	inhibit ferroptosis and restore synaptic plasticity	Depression↓	Berezutsky et al. (2022)
Catalpol	Mice	ATF3/FSP1 signalling mediates ferroptosis	Promoting antidepressant effect of fluoxetine↓	Wu et al. (2024a)
Cynaroside	CUMS mice	IRF1/SLC7A11/GPX4↑	Depression↓	Zhou et al. (2024)
Silybin	Mice	p53↓and SLC7A11↑GPX4↑STING↓	Neuroinflammation↓	Dai et al. (2023)
Lycium barbarum glycopeptide	Chronic restrain stress mice	IPLA2, GPX4↑,4-HNE and MDA↓	Anxiety and depressive behaviour↓	Ran et al. (2024)
XiaoYao San	CUMS mice	Activation of PEBP1-GPX4	Depression↓	Jiao et al. (2021)
Di-Huang-Yin-Zi	PSD rat	ROS, MDA, Fe^2+^↓; SLC7A11 and GPX4↑ promotes P53	Depression↓	Long et al. (2024)
SuanZaoRen decoction	APP/PS1 mice	iron↓TfR1, FTH1, FTL and ACSL4 proteins↓ FPN1, DJ-1, Nrf2, GPX4 and SLC7A11↑	Inhibit neuron loss, synaptic damage	Du et al. (2024b)

OVX: ovariectomy; STING: stimulator of interferon gene; 4-HNE: 4-Hydroxynonenal; FTH1: Ferritin Heavy Chain 1; FTL:ferritin light chain; ACSL4:Long-chain acyl-CoA, synthetase 4; FPN1:Ferroportin 1.

### 9.5 Chinese medicine targeting ferroptosis for treatment of atherosclerosis co-depression

The preceding discussion highlights various monomers and compounds that target ferroptosis for treating AS and depression. Certain pharmacological agents, despite their differences, may share common mechanisms in targeting ferroptosis. For example, PAE not only protects against AS but also exerts neuroprotective effects by inhibiting neuronal ferroptosis in cerebral hemorrhage (Jin et al., 2021) and serves as an antidepressant (Wei et al., 2023). HSYA, known for its excellent blood-brain barrier permeability, improves depressive behavior by inhibiting hippocampal inflammation and oxidative stress (Liu Z. et al., 2022). Similarly, Icariin has shown promising antidepressant effects (Zeng et al., 2022). Quercetin also exhibits the potential to inhibit atherosclerotic ferroptosis while serving as an antidepressant by targeting ferroptosis-related mechanisms (Li H. et al., 2024). CNS inhibits the abnormal proliferation of aortic vascular smooth muscle cells (Kim et al., 2006). In summary, TCM effectively treats AS-related depression by targeting ferroptosis, which is also an important therapeutic target.

## 10 Conclusions and future perspectives

In conclusion, studying the mechanisms of comorbidity through the perspective of ferroptosis is essential, with GPX4 and Nrf2 being highlighted as critical targets. Cellular focus includes macrophages, endothelial cells, microglia, and neurons. Regarding treatment, Chinese medicine, known for its multi-target effects, holds significant potential. Ferroptosis not only directly contributes to AS but also interacts with inflammatory responses, energy metabolism, and the gut, accelerating disease progression. Targeting ferroptosis offers a promising therapeutic approach for AS and depression, providing a new strategy for addressing comorbidity. We highlighted the role of ferroptosis in comorbidities and emphasized its relevance in cardio-cerebrovascular diseases associated with AS and psychiatric disorders, such as depression. This underscores the broader significance of the ferroptosis mechanism across various diseases and provides insights into its involvement in other conditions.

Despite significant progress in understanding ferroptosis in AS and depression, its role in comorbid conditions remains unclear. In the clinical treatment of patients with atherosclerotic co-depressive disorders, combining lipid-lowering drugs and antidepressants often causes side effects such as nausea, dizziness, and vomiting, resulting in treatment failure. Therefore, identifying shared pathogenesis is crucial, and targeting ferroptosis represents a promising strategy. For example, deferoxamine, an iron-chelating agent, is an approved treatment for iron overload. It improves endothelium-dependent vasodilation in patients with coronary heart disease (Duffy et al., 2001), protects neurons from ferroptosis, and exhibits antidepressant effects (Trachtenberg et al., 2011). However, deferoxamine can cause growth delay, allergic reactions and bone abnormalities, and at high doses, it can cause nervous system damage (Entezari et al., 2022). Despite challenges such as poor solubility and instability, many active ingredients in Chinese medicine offer multi-target effects and lower toxicity, making them a preferable option.

TCM plays a significant role in treating atherosclerotic co-depression diseases through its anti-ferroptosis effects, but the specific molecular targets remain unclear. TCM consists of numerous components; however, only a few are absorbable, which complicates the understanding of their mechanisms and limits the scientific credibility of TCM. Anti-AS co-depression drugs targeting ferroptosis also have side effects. For example, thiazolidinediones, which inhibit ACSL4, can cause cardiac toxicity with prolonged use (Al Sultan et al., 2024), while zileuton, a lipoxygenase inhibitor, may lead to indigestion, nausea, and other issues. Although ferroptosis has been implicated in these diseases, its exact role in the occurrence and progression of comorbidities during specific pathogenesis remains unclear. The mechanism of ferroptosis in comorbidities is not fully understood. These limitations highlight the direction of our efforts in studying comorbidity.
